# TaIlored ManagEment of Sleep (TIMES) for people with dementia/mild cognitive impairment – A Cluster Randomised Feasibility Trial

**DOI:** 10.1002/alz70858_099722

**Published:** 2025-12-24

**Authors:** Chris Fox, Jayden O van Horik, Louise M Allan, Andrea Hilton, Anne Killett, Gill Livingston, Ian D Maidment, Antonieta Medina‐Lara, Joanne Reeve, Geoff Wong

**Affiliations:** ^1^ University of Exeter, Exeter, Devon, United Kingdom; ^2^ University of Hull, Hull, United Kingdom; ^3^ University of East Anglia, Norwich, United Kingdom; ^4^ University College London, London, London, United Kingdom; ^5^ Aston University, Birmingham, United Kingdom; ^6^ Hull York Medical School, University of Hull, Hull, United Kingdom; ^7^ Oxford University, Oxford, United Kingdom

## Abstract

**Background:**

The increasing global direct and indirect cost of dementia care reached 1.33 trillion USD in 2020 with a projected rise to 9.12 trillion USD by 2050. Sleep disturbances are prevalent in People Living With Dementia (PLWD) and Mild Cognitive Impairment (MCI), impacting on daily living, increasing carer burden, and potentially accelerating disease progression. Effective sleep management could improve independence, reduce carer burden, and mitigate long‐term economic costs. However, little is known about how to effectively manage sleep in PLWD/MCI within primary care settings. Challenges stem from a complex interplay of contributing patient and practice factors including inappropriate long‐term hypnotic usage, diverse individual experience and perception of needs, and limited access to effective non‐pharmacological interventions. Effective sleep management for PLWD/MCI might however be improved with tailored, person‐centred, care plans with the input of family carers, that address individual needs and circumstances and promote independence.

**Methods:**

We co‐designed a Tailored Intervention for Managing Sleep (TIMES) with Patient and Public Involvement (PPI) groups and health and social care professionals. TIMES aims to support General Practitioners and other primary care staff, e.g. nurses, pharmacists, to provide person‐centred care for PLWD and MCI, by addressing the multifaceted aspects of sleep disturbance. In preparation for evaluating the effectiveness of TIMES in a subsequent, full‐scale, randomised controlled trial, we conducted a cluster‐randomized feasibility trial in 10 GP practices in England, targeting 64 patient‐carer dyads, including economic analysis.

**Results:**

We will present key findings from the feasibility trial, highlighting (i) barriers and enablers to successful recruitment and retention of both GP practices and patient‐carer dyads; (ii) intervention fidelity and acceptability within the primary care setting; (iii) preliminary outcomes of the TIMES intervention on patient sleep, quality of life, carer burden, costs and resource utilisation including medication, and primary care staff confidence in managing sleep disturbances in PLWD/MCI.

**Conclusions:**

We will discuss the implications of our findings for improving sleep management for PLWD/MCI within primary care settings. Findings from this feasibility trial will inform the design and deployment of a subsequent full‐scale randomized controlled trial of the TIMES intervention.

